# Assessment of SARS-CoV-2 immune escape using antigenic cartography combined with experimental challenge studies

**DOI:** 10.1038/s41541-025-01348-x

**Published:** 2026-01-02

**Authors:** Merel R. te Marvelde, Anna Z. Mykytyn, Edwin J. B. Veldhuis Kroeze, Alexandre H. J. Bouffier, Debby van Eck—Schipper, Petra van den Doel, Kim Handrejk, Björn Koel, Melanie Rissmann, Bart L. Haagmans

**Affiliations:** Department of Viroscience, Erasmus Medical Center, Rotterdam, The Netherlands

**Keywords:** Diseases, Immunology, Microbiology

## Abstract

The disease burden of COVID-19 significantly decreased with the implementation of vaccines. However, SARS-CoV-2 variants that escape vaccine induced immunity continue to emerge and may pose a risk to public health. While vaccine updates are available, it remains uncertain whether they are required for full protection. Here, we antigenically characterized SARS-CoV-2 variants JN.1, KP.2, KP.3.1.1, XEC and LP.8.1 by antigenic cartography and evaluated in vivo protection of JN.1 vaccination in hamsters. Antigenic cartography revealed that these variants are antigenically closely related. In vivo experiments showed that JN.1 vaccination blocked viral replication and inflammation in the lower respiratory tract of JN.1, KP.2 and KP.3.1.1 infected animals. However, despite close antigenic proximity, KP.3.1.1 infected JN.1 vaccinated animals showed evidence of viral replication in the upper respiratory tract, indicative for immune escape. These data demonstrate the strength of combining antigenic cartography with experimental challenge studies to study SARS-CoV-2 immune escape for vaccine updates.

## Introduction

Emerging SARS-CoV-2 variants have evolved continuously after the introduction of SARS-CoV-2 vaccines. With the emergence of Omicron variants in autumn 2021, immune evasion was identified as a major driving force of the evolution of SARS-CoV-2, enabling the virus to sustain high transmissibility and cause reinfections in immune populations^1^. Immune evasion of JN.1 lineages from the previous vaccine strain XBB.1.5^2–6^, led to the WHO recommendation to update vaccines towards JN.1 to induce protection and prevent severe disease and hospitalization^7^. Therefore, vaccination campaigns with the newly authorized monovalent COVID-19 mRNA vaccine based on the spike protein of JN.1 (Europe) and its subvariant KP.2 (USA) were started in 2024. However, continuous immune pressure for SARS-CoV-2 has resulted in emergence of new variants like KP.3.1.1, XEC and LP.8.1. SARS-CoV-2 vaccine updates aim to sustain population immunity against those newly emerging variants. It is important to assess whether current vaccines are able to prevent transmission and protect against severe disease.

Therefore, there is a need to further develop and combine experimental approaches to validate antigenic differences of emerging SARS-CoV-2 variants in detail. A tool to investigate and visualize immune evasion and antigenic relationships between viral strains, providing valuable insights for vaccine strain selection, is antigenic cartography^8^. Previously generated SARS-CoV-2 antigenic maps have demonstrated immune escape of several Omicron variants^9–11^ and antigenic cartography contributed to decisions on vaccine updates^7^. Several maps have investigated the positioning of newly emerging variants JN.1, KP.2 and KP.3.1.1, indicating that these form a separate group from XBB.1.5 and earlier SARS-CoV-2 variants^12,13^. However, our understanding how antigenic differences observed in antigenic maps translate to escape from vaccine induced immunity is limited. In addition, to answer the question how well JN.1 vaccination protects from currently relevant variants and if a future vaccine update is necessary, more recent SARS-CoV-2 variants such as XEC and LP.8.1 should be included in the map.

The first studies reporting JN.1 vaccine induced immune responses in humans have shown that vaccination with JN.1 can increase neutralizing antibody titers against JN.1, KP.3.1.1, and XEC^14–17^. Although the reduced height of KP.3.1.1, XEC, and LP.8.1 neutralizing antibody titers after vaccination compared to JN.1 homologous titers might imply partial immune escape^16,18,19^. Similar increases in humoral immunity have been seen after breakthrough infection with JN.1^20,21^ and after vaccination with KP.2^22^. Humoral immune responses are a correlate of protection for COVID-19 disease caused by SARS-CoV-2^23^, but ultimately the goal of vaccination is to confer protection from (severe) disease and limit transmission. The first vaccine effectiveness (VE) estimations showed that vaccination with a 2024-2025 COVID-19 vaccine dose (containing JN.1 or KP.2) provides additional protection against hospitalization compared to not receiving a 2024-2025 dose^24^, indicating the relevance of waning immunity and necessity of booster vaccinations. However, this data lacks variant specific analysis and potentially has a lot of confounding factors like prior vaccines received, previous SARS-CoV-2 infections and comorbidity which can impact the VE estimates. Therefore, it is essential to verify vaccination induced protection against replication in the upper- and lower respiratory tract in experimental animal models, overcoming those confounding factors.

Ideally, verification of protection and efficacy conferred by a vaccine is evaluated in a controlled system where pathogenesis, infectious viral titers in relevant target tissues and immune responses can be measured. Golden Syrian hamsters are a reliable animal model for SARS-CoV-2 infections, since they mimic disease progression and pathology as seen in humans^25^ and can be utilized to specifically study immunogenicity and efficacy of new vaccines in a pre-clinical setup.

To assess the necessity of vaccine updates, a predictive model is needed to estimate whether and when a vaccine update would enhance immunity and protect the majority of the population from disease. In this study, we aim to evaluate the cross-protection induced by JN.1 spike vaccination against recently circulating variants by creating an updated antigenic map. Additionally, we verify the predictive value of the antigenic map by evaluating the in vivo protection provided by JN.1 spike vaccination against JN.1, KP.2, and KP.3.1.1 replication in upper- and lower respiratory tract in a golden Syrian hamster model.

## Results

### Recently circulating SARS-CoV-2 variants induce strong humoral immune responses to JN.1, KP.2, KP.3.1.1, XEC and LP.8.1

**Fig. 1:**
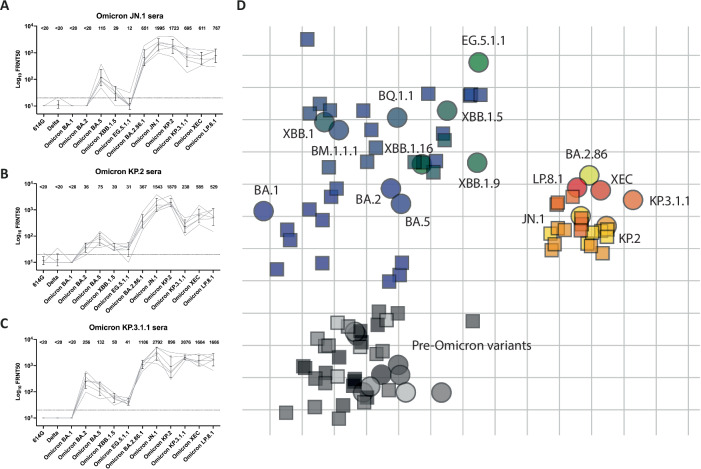
Antigenic characterization of recently circulating SARS-CoV-2 variants reveals their close antigenic relationship to JN.1. Hamsters were challenged with (**A**) JN.1, (**B**) KP.2 or (**C**) KP.3.1.1. Serum samples were collected 14 dpi. FRNT_50_ titers were determined against a range of SARS-CoV-2 variants (X-axis). Individual values (grey line) and geometric mean titers (black line) with 95% CI are shown. The dotted line indicates the lower limit of detection. **D** Antigenic map showing SARS-CoV-2 antigens (circles) and corresponding antisera (squares). One grid distance in the map corresponds to a 2-fold difference in neutralizing titers. Pre-omicron variants are depicted in shades of grey, early-Omicron variants up to EG.5.1.1 are depicted in shades of blue and green, and recently circulating variants are depicted in shades of yellow and red. Antisera are colored the same as their corresponding SARS-CoV-2 antigen.

To evaluate neutralizing antibody responses after infection with recently circulating variants, we examined the immune responses 14 days post inoculation (dpi) with JN.1, KP.2, or KP.3.1.1 in (mock-vaccinated) hamsters (Fig. 1). All three variants induced similar patterns of neutralizing antibody responses with high neutralizing antibody titers against the homologous variant for each of the three variants. None of the variants was able to induce significant neutralizing antibody responses against 614 G, Delta and BA.1 and they induced no or low neutralizing antibodies against BA.2, BA.5, XBB.1.5 and EG.5.1.1 (Fig. 1A-C). Interestingly, KP.3.1.1 infection yielded the most cross-neutralizing antibodies with the highest titers against the recently emerged XEC and LP.8.1, while inducing high antibodies against the previously circulating BA.2.86.1 (Fig. 1C). Although JN.1 and KP.2 infection also induced neutralizing antibody titers against these variants, they were noticeably lower (Fig. 1A, B).

### Antigenic cartography reveals the broad protective spectrum of JN.1 induced antibodies to recently circulating variants

Next, we investigated the antigenic relations of the recently circulating variants compared to earlier SARS-CoV-2 variants by creating an antigenic map. Previously generated sera from hamsters inoculated with SARS-CoV-2 variants 614G, Delta, Omicron BA.1, Omicron BA.5, Omicron BM.1.1.1, and Omicron XBB.1.5^10^ were used to determine the neutralizing titer against new Omicron variants JN.1, KP.2, KP.3.1.1, XEC, and LP.8.1 (Supplementary Fig. 1A-E). All neutralizing titers of previous hamster antisera and new hamster antisera of the different SARS-CoV-2 variants were combined to create an update of the SARS-CoV-2 antigenic map (Fig. 1D). The antigenic map shows close clustering of Pre-Omicron variants. Early Omicron variants like BA.1, BA.2, BA.5, and XBB.1.5 grouped separately from these Pre-Omicron variants and occupied a widespread antigenic space on the map. The more recently circulating Omicron variants BA.2.86.1, JN.1, KP.2, KP.3.1.1, XEC, and LP.8.1 group closely together, separate from the previously mentioned groups. The antisera of the recently circulating variants are positioned close to the position of the viruses. This indicates that antisera generated by infection with either of these variants can neutralize viruses from this group relatively well. Specifically, the positioning of JN.1 sera on the antigenic map indicates that JN.1 vaccination could induce cross-protection to newly circulating variants KP.2, KP.3.1.1, XEC, and LP.8.1. Triangulation appeared to be low (Supplementary Fig. 2A) and the map distance correlated to the table distance (Supplementary Fig. 2B). To verify that the two-dimensional map provides an accurate representation of the data, a three-dimensional map was made (Supplementary Fig. 2C-D). Dimensionality testing (Supplementary Fig. 2E) and comparison to the two-dimensional positioning (Supplementary Fig. 2F) showed in general accurate and representative placement of the antigen and antisera on the two-dimensional map.

### JN.1 vaccination reduces viral shedding after challenge with SARS-CoV-2 JN.1, KP.2 and KP.3.1.1

**Fig. 2:**
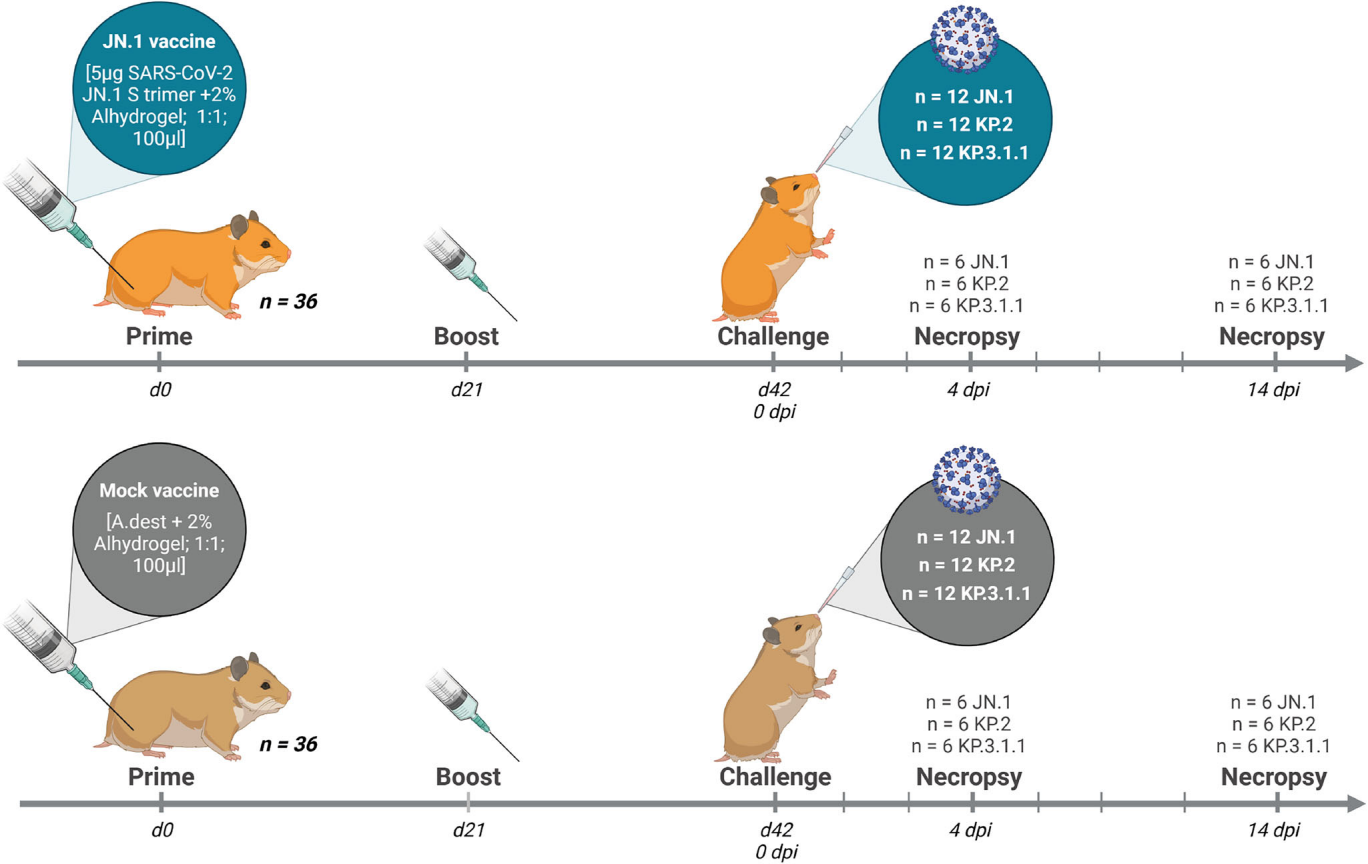
Experimental setup of JN.1 vaccination. Experimental setup of in vivo evaluation of JN.1 vaccine efficacy against challenge with JN.1, KP.2 and KP.3.1.1. Golden Syrian hamsters were primed with JN.1 or a mock vaccine and received a booster after 21 days. On day 42 the hamsters were challenged with Omicron variants JN.1, KP.2 or KP.3.1.1. Throat swabs and collective feces samples per cage were taken at 1, 3, 5, 7, 10 and 14 days post inoculation (dpi). At 4 and 14 days post inoculation (dpi), animals were sacrificed to determine the viral load in tissue and blood was collected for serological analysis. Created in BioRender. 2, V. (2025) https://BioRender.com/13issf0.

To evaluate whether the results from the antigenic analysis translate into in vivo protection against infection, we determined the protective efficacy of a JN.1 spike vaccination against challenge with SARS-CoV-2 JN.1, KP.2, and KP.3.1.1 in a hamster model (Fig. 2).

**Fig. 3:**
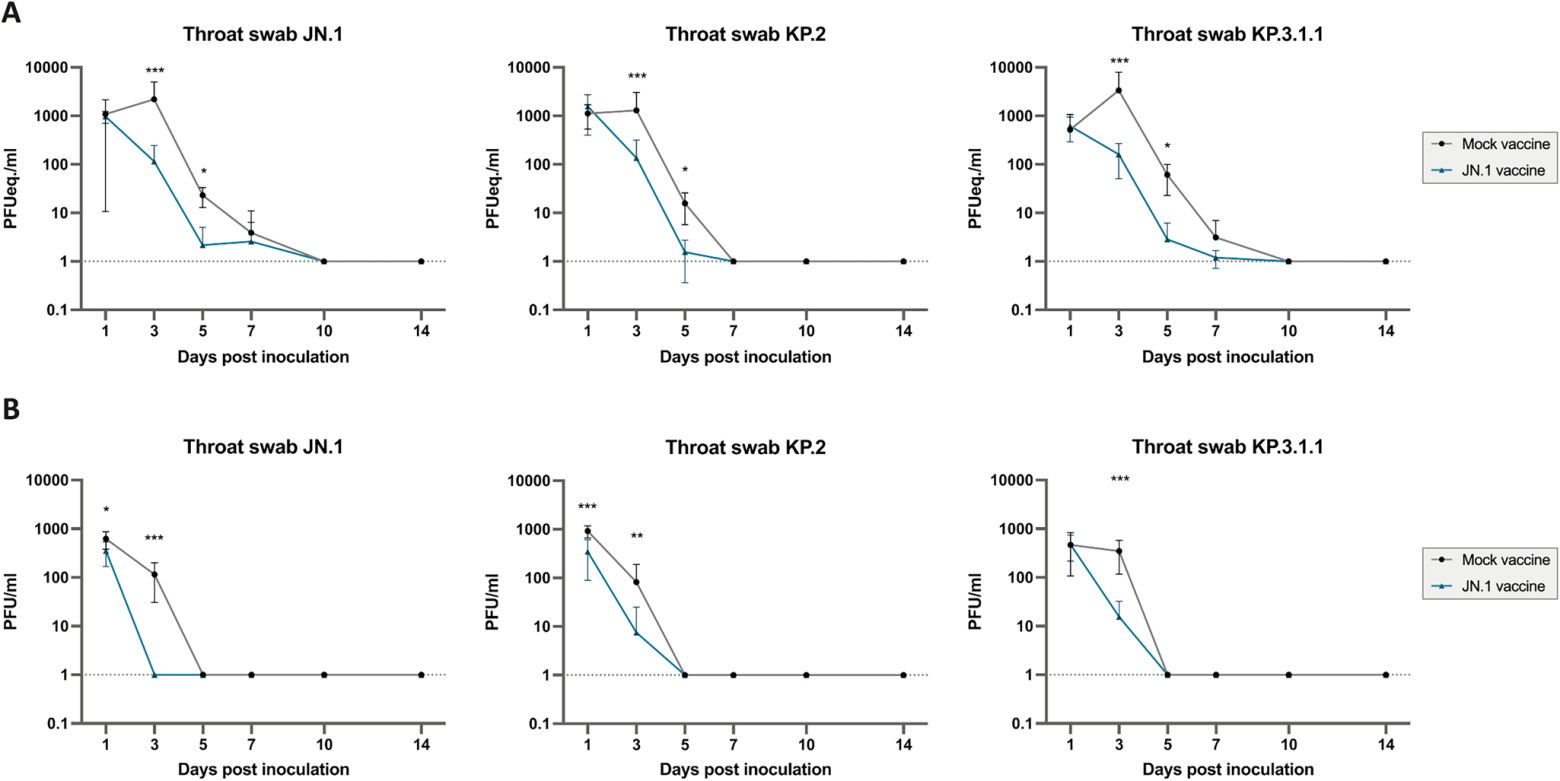
JN.1 vaccination reduces viral shedding after challenge with SARS-CoV-2 JN.1, KP.2 and KP.3.1.1. **A** RT-qPCR results of throat swabs, depicted in PFUeq./ml. **B** Results of plaque assay to determine infectious virus in PFU/ml. Statistical significant differences were measured by Mann-Whitney test with multiple comparisons according to the Holm-Šídák method. Error bars represent interquartile range. *p < 0.05, **p < 0.005, ***p < 0.001.

Both mock-vaccinated and JN.1 vaccinated animals did not show adverse clinical signs or changes in the relative body weight during the experiment (Supplementary Fig. 3A). A significant JN.1 vaccine-induced reduction of viral shedding was observed (Fig. 3). Independent of the challenge virus, viral genome copies in throat swabs were significantly reduced on 3 and 5 dpi (Fig. 3A) (JN.1 challenge, p < 0.0001 on 3 dpi and p = 0.011 on 5 dpi; KP.2 challenge, p = 0.00087 on 3 dpi and p = 0.021 on 5 dpi; KP.3.1.1 challenge, p < 0.0001 on 3 dpi and p = 0.011 on 5 dpi). Furthermore, JN.1 vaccination induced significant decrease in infectious virus shedding of all challenge viruses (Fig. 3B) (JN.1 challenge, p = 0.032 on 1 dpi and p < 0.0001 on 3 dpi; KP.2 challenge, p = 0.00039 on 1 dpi and p = 0.0054 on 3 dpi; KP.3.1.1 challenge, p < 0.0001 on 3 dpi). However, an indication of extended shedding of infectious virus was observed on 3 dpi for JN.1 vaccinated animals that were challenged with KP.2 and KP.3.1.1, when compared to JN.1 challenged animals. Limited viral genome was detected in bulk fecal samples with slight differences between JN.1 and mock-vaccinated and KP.3.1.1. challenged animals (Supplementary Fig. 3B).

### Protection of the lower respiratory tract against viral replication and reduced inflammation after JN.1, KP.2, and KP.3.1.1 challenge achieved through JN.1 vaccination

**Fig. 4:**
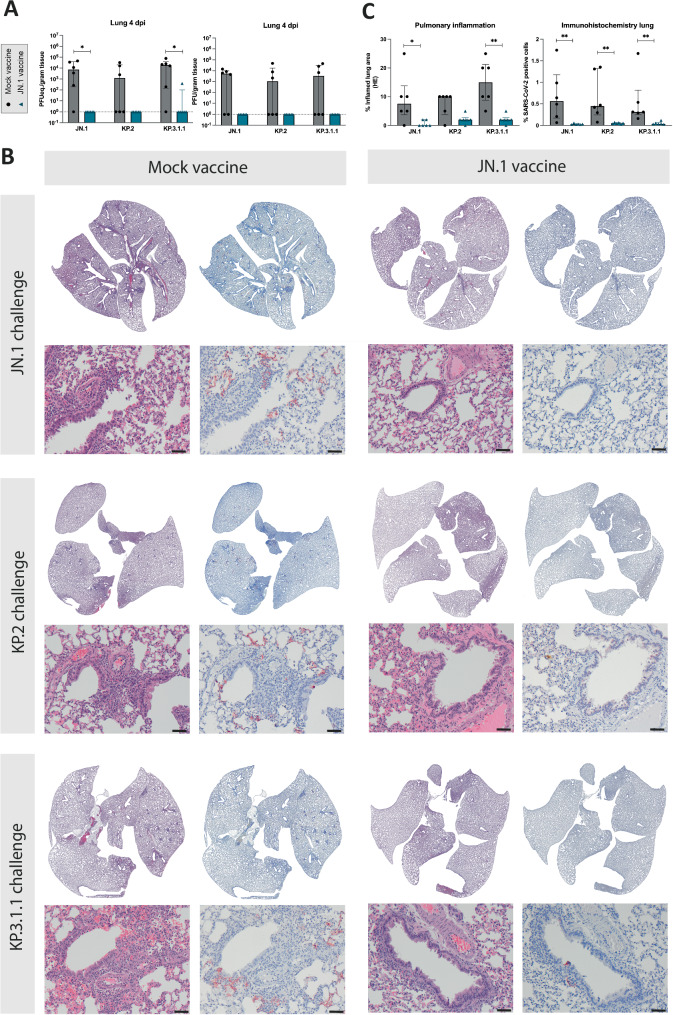
Protection of the lower respiratory tract against viral replication and reduced inflammation after JN.1, KP.2, and KP.3.1.1 challenge achieved through JN.1 vaccination. **A** RT-qPCR results of respiratory tissue homogenates depicted in PFUeq./g tissue and results of plaque assay to determine infectious virus in PFU/g tissue. Statistical significant differences were measured by Mann-Whitney test. Error bars represent interquartile range. *p < 0.05, **p < 0.005. **B** Histopathology panel showing H&E and corresponding serial immunohistochemistry (IHC) lung slides at 4 dpi (days post infection) of mock-vaccinated hamsters compared to JN.1-vaccinated hamsters following intranasal challenge with one of three SARS-CoV-2 variants JN.1, KP.2, and KP.3.1.1, from top to bottom, respectively. Virus antigen expression is notably present co-localized with inflammatory lesions and consolidated areas of lung parenchyma in mock-vaccinated animals, compared to notably less affected lung parenchyma in JN.1 vaccinated animals when similarly challenged. Stained by hematoxylin and eosin (H&E); and virus antigen is expressed as reddish-brown staining of alveolar and/or bronchial epithelial cells’ cytoplasm by AEC-immunoperoxidase, on hematoxylin counterstain. Whole lung slide scans and original magnifications of 100×. Scale bars indicate 50 µm. **C** Quantification of relative inflamed area of lungs, as evaluated by a board-certified veterinary pathologist and relative SARS-CoV-2 antigen positive area in IHC, quantified with positive cell detection threshold in QuPath Open software version 0.3.2. Statistical significant differences were measured by Mann-Whitney test. Error bars represent interquartile range. Symbols represent individual measurements for each animal. *p < 0.05, **p < 0.005. Six animals were included per experimental condition.

Vaccination eliminated viral replication in the lungs of all JN.1 vaccinated animals regardless of challenge virus, although differences of viral genome and infectious virus compared to mock-vaccinated animals were partially not significantly different due to high variability within experimental groups (Fig. 4A). Because of limited recovery of infectious virus in the trachea, differences were less striking in this tissue but were still evident comparing the viral genomic load without obvious differences between different challenge viruses (Supplementary Fig. 4A) (JN.1, KP.2 and KP.3.1.1. challenge, p = 0.0022 for viral genome). No infectious virus was detected in trachea or lung on 14 dpi. (Supplementary Fig. 4B**)**.

On 4 dpi, the lungs of the mock-vaccinated groups showed an acute inflammation that was on average mild in severity and extent, and multifocal centered on bronchioles. It was characterized by consolidated alveolar parenchyma (Fig. 4B) due to swollen septa and exudates of neutrophils and edema, mixed with macrophages and erythrocytes. Cytoplasmic virus antigen expression co-localized with these inflamed foci affecting both epithelia lining the alveoli and bronchioles. In comparison to mock-vaccinated animals, all three JN.1 vaccinated and challenged groups showed on average a similar but partially significantly reduced pulmonary inflammation regardless of the different challenge virus strains (JN.1 challenge, p = 0.020; KP.3.1.1 challenge, p = 0.0043), and significantly reduced antigen expression (JN.1 challenge, p = 0.0022; KP.2 challenge, p = 0.0022; KP.3.1.1 challenge, p = 0.0022) (Fig. 4C). No virus antigen expression was observed in the tracheas on 4 dpi in all animals (Supplementary Table 1). The overall cytokine induction in the lungs of vaccinated and challenged hamsters was limited and did not show significant differences between vaccination regimes (Supplementary Fig. 5).

### Reduced viral replication and inflammation in the upper respiratory tract after JN.1 vaccination

**Fig. 5:**
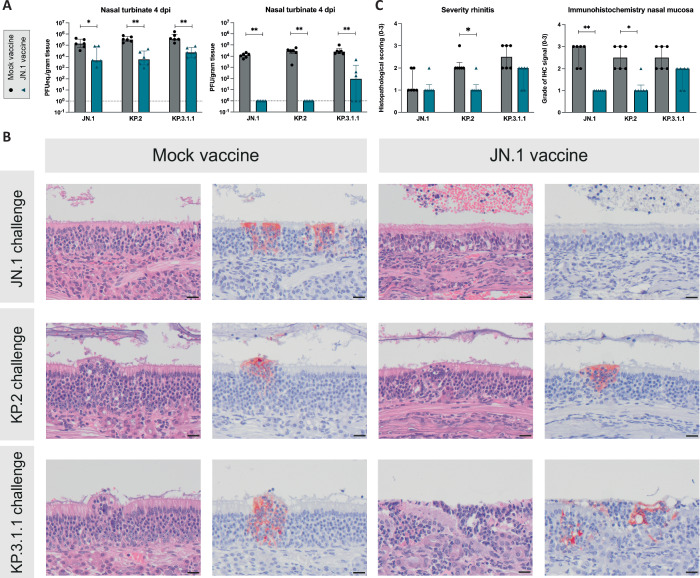
Reduced but remaining viral replication in the upper respiratory tract after JN.1 vaccination and KP.3.1.1 challenge. **A** RT-qPCR results of respiratory tissue homogenates depicted in PFUeq./g tissue and results of plaque assay to determine infectious virus in PFU/g tissue. Statistical significant differences were measured by Mann-Whitney test. Error bars represent interquartile range. *p < 0.05, **p < 0.005. **B** Histopathology panel showing H&E and corresponding serial immunohistochemistry (IHC) slides of nasal olfactory mucosa at 4 dpi of JN.1-vaccinated hamsters compared to mock-vaccinated hamsters following intranasal challenge with one of three virus variants JN.1, KP.2, and KP.3.1.1, from top to bottom respectively. Virus antigen expression is present that co-localizes with inflammatory lesions in all animals’ nasal mucosa, albeit to a slight reduced averaged score in vaccinated groups compared to mock-vaccinated groups (not evident from these close-up magnifications). Stained by hematoxylin and eosin (H&E); and virus antigen is expressed as reddish-brown staining of epithelial cells’ cytoplasm by AEC-immunoperoxidase, on hematoxylin counterstain. Original magnifications 200×. Scale bars indicate 20 µm. **C** Histopathological scoring of rhinitis and grade of IHC signal in the nasal mucosa, as evaluated by a board-certified veterinary pathologist. Statistical significant differences were measured by Mann-Whitney test. Error bars represent interquartile range. Symbols represent individual measurements for each animal. *p < 0.05, **p < 0.005. Six animals were included per experimental condition.

Additionally, vaccination had a significant effect on viral replication in the upper respiratory tract 4 dpi (Fig. 5A). JN.1 vaccinated animals showed a significant reduction of viral genome and infectious virus in the nasal turbinate (JN.1 challenge, p = 0.026 for viral genome and p = 0.0022 for infectious virus; KP.2 challenge, p = 0.0022 for viral genome and p = 0.0022 for infectious virus; KP.3.1.1 challenge, p = 0.0022 for viral genome and p = 0.0022 for infectious virus). Noticeably, prevention of viral replication was least efficient in KP.3.1.1 challenged animals, where replicating virus was detected in the nasal turbinate of three out of six JN.1 vaccinated animals, indicating partial protection of the JN.1 vaccine for KP.3.1.1 in the upper respiratory tract. Limited viral genome was found in the nasal turbinate on 14 dpi with significant differences between mock- and JN.1 vaccinated animals that were challenged with JN.1 and KP.3.1.1 (Supplementary Fig. 6A) (JN.1 challenge, p = 0.041; KP.3.1.1. challenge, p = 0.0087). No infectious virus was detected in the nasal turbinate 14 dpi.

On 4 dpi, the nasal mucosa of the mock-vaccinated groups showed an acute inflammation that was on average mild to moderate in severity, multifocal distributed, and characterized by infiltration of mainly neutrophils in both the epithelial lining and in underlying glands, including Bowmans glands. Affected epithelial cells were swollen or were necrotic and sloughed. The nasal passages contained serous exudates with neutrophilic and epithelial cellular debris (Fig. 5B). Cytoplasmic virus antigen expression co-localized with these inflamed foci affecting both the respiratory and olfactory mucosae, with a slight bias towards the olfactory mucosa in most animals. In comparison, JN.1-vaccinated and challenged groups showed a similar but reduced rhinitis and reduced virus antigen expression that were slight or mild in severity regardless of the different challenge virus strains, when compared to mock-vaccinated animals (Fig. 5C, Supplementary Fig. 6B).

### Extra respiratory tissues and serum confirm protective JN.1 vaccination effects

A clear protection from viral replication was furthermore observed in non-respiratory tissue. While no viral genome or infectious virus was detected in small- and large intestines, a clear vaccine-induced reduction of viral genome was observed in the olfactory bulb on 4 dpi, that was independent of challenge virus (Supplementary Fig. 7A) (JN.1, KP.2 and KP.3.1.1. challenge, p = 0.0022 for viral genome). On 14 dpi, no evidence of viral replication was detectable in extra-respiratory tissues (Supplementary Fig. 7B). In none of the animals virus antigen expression was observed in the central nervous tissue (CNS) nor in the intestines at 4 dpi (Supplementary Table 1).

While all animals were vaccinated with JN.1, variable challenge strains were used to investigate differences in antibody responses seen after challenge. On 14 dpi, induced humoral immunity was assessed by measuring the neutralizing antibody responses against the viruses JN.1, KP.2, and KP.3.1.1. Hamsters that received the JN.1 vaccine prior to JN.1 infection showed increased neutralizing antibody titers for JN.1, KP.2, and KP.3.1.1 after JN.1 challenge. After KP.2 challenge, JN.1 and KP.3.1.1 neutralizing antibodies were increased for vaccinated hamster compared to their mock-vaccinated controls, but KP.2 neutralizing antibodies showed a slight decrease. Similarly, after KP.3.1.1 challenge, JN.1 and KP.2 titers increased in vaccinated animals, but KP.3.1.1 showed a decrease (Supplementary Fig. 8). To investigate the potential effect of antigenic imprinting caused by vaccination with the JN.1 trimer, we compared the titers against the primary exposure antigen with the challenge virus. JN.1 vaccinated KP2 and KP.3.1.1 challenged animals showed higher titers against the primary antigen JN.1 than against the challenge antigen, suggesting a possible imprinting effect, although the differences were not significant. Overall, these data indicate that vaccination with JN.1 enhances neutralizing antibody titers after infection, resulting in a general increase in titers compared to mock-vaccinated animals. Combined with the lower titers of virus in nasal turbinate, trachea and lungs these results show a protective effect of JN.1 vaccination for infection with JN.1, KP.2, and KP.3.1.1.

## Discussion

With the continuous emergence of SARS-CoV-2 variants, it is uncertain whether vaccine updates are necessary, and which strain should be used for immunization. Aggravating factor in the decision making of vaccine strategies is that we currently cannot precisely define correlates of protection against severe disease or transmission of newly emerging variants. Lower neutralizing antibody titers are an indication of immune evasion and often serve as a basis for next vaccine strain selection. However, the magnitude of immune evasion that leads to disease and foremost transmission remains elusive. Here, we sought to determine the antigenic relationships between recently circulating SARS-CoV-2 variants and use this information to predict vaccine efficacy of JN.1 vaccination. In line with estimations of the antigenic map, a JN.1 spike vaccine induced protection from disease against JN.1, KP.2, and KP.3.1.1 in vivo. However, despite only marginal immune evasion of KP.3.1.1, limited replication in the upper respiratory tract was detected.

In agreement with other recently published antigenic maps, we observe the separation and grouping of the JN.1 derived variants in a relatively small antigenic space^12,13^, indicating that the JN.1 vaccine could induce cross-protection against these variants. Our antigenic map additionally shows the positioning of KP.3.1.1 antisera, and antigens KP.3.1.1, XEC, and LP.8.1. Although KP.3.1.1 appears to be the most distant of the cluster with JN.1 variants, it remains within two antigenic distance units, suggesting that there is limited immune evasion, corresponding to findings from others investigating human sera, indicating limited immune evasion of KP.3.1.1^4,14–18,20^. Additionally, the antigenic map confirms significant immune escape of JN.1 from the previous vaccine strain, XBB.1.5, as also observed in humans after vaccination and breakthrough infection^5,6^ and using antigenic cartography^12,13,26^. To accurately determine antigenic relationships between SARS-CoV-2 variants and track antigenic evolution, it is important to use single exposure sera, as variable population immunity around the world and the confounding effects of pre-existing immunity may impact the structure of the antigenic map. Therefore, we use golden Syrian hamster sera, as our and others findings indicate that hamster humoral immune responses closely resemble human responses, resulting in comparable antigenic maps^9,26,27^. However, pre-existing hybrid immunity in the human population may influence the response to new antigens through imprinting, depending on the vaccination and infection history of an individual^28,29^. Overall, in vivo efficacy testing of SARS-CoV-2 vaccines in golden Syrian hamsters combined with antigenic mapping using hamster sera complements studies using sera from humans to our further understanding of the antigenic relationship among SARS-CoV-2 variants.

Virus replication in the upper and lower respiratory tract, evident by presence of viral genome, infectious virus, and viral antigen was observed in mock-vaccinated hamsters independent of challenge virus. In contrast to observations of other groups, failing to rescue infectious virus after JN.1 infection using Vero cells^30^, human lung adenocarcinoma cells (Calu-3) were applied in this study and highlight the necessity of relevant cell models for viral detection and propagation. Although titers are markedly reduced when compared to earlier variants, results underline the applicability of the current hamster model to evaluate efficacy of updated vaccination strategies against newly emerging variants of SARS-CoV-2. Shedding of infectious virus was significantly reduced as detected in throat swabs of vaccinated and challenged animals, with indications of prolonged shedding in KP.2 and KP.3.1.1 challenged animals. While results would need confirmation in in vivo transmission experiments, an effect on transmission is indicated and therefore underlines the beneficial effect of a vaccine-update for global public health. Additionally, the JN.1 spike vaccination fully prevented viral replication in the upper respiratory tract in JN.1 and KP.2 challenged animals. However, a decrease of protection was observed for three out of six KP.3.1.1 infected animals indicated by remaining presence of viral antigen and infectious virus in the upper respiratory tract and being in line with observations of extended shedding of KP.3.1.1 challenged animals. Comparable to previous reports^31^, variants being antigenically more distinct to the vaccine antigen as derived from the antigenic analysis, might partially escape immunity, allowing limited replication in the nasal cavity, whereas protection in the lower respiratory tract is warranted. Observed indications of immune escape by KP.3.1.1 might be driven by mutations enabling glycosylation, which shields antibodies from binding to the spike protein^32^. Recent studies have demonstrated that KP.3.1.1 and XEC have mutations in the N-terminal domain introducing a new glycosylation site that might impact immune evasion of these variants, possibly by reducing the binding of neutralizing antibodies to the receptor-binding domain through allosteric mechanisms^33,34^. Together, these observations are essential to prospectively frame the development of genotype-to-phenotype pipelines, including predictions of antigenicity and vaccine-induced protection.

Despite reduced protection in KP.3.1.1 challenged animals in the upper respiratory tract, viral replication in the lower respiratory tract was entirely prevented by JN.1 vaccination for all three challenge viruses, indicating the protection from severe disease. Results were consolidated by histopathological findings, where an acute mild bronchitis and alveolitis, accompanied by cytoplasmic viral antigen were observed in mock-vaccinated groups. Pulmonary inflammation and evidence of viral antigen was significantly reduced in vaccinated animals, irrespective of challenge virus. In summary, a clear vaccine mediated protection from viral replication in the lower respiratory tract and induced disease can be observed for all three challenge viruses, as predicted by antigenic proximity of vaccine and challenge antigens. Even though in this study we did not investigate the response of T cells, T cell epitopes are known to be generally more preserved between SARS-CoV-2 variants^35^, possibly contributing to the strong protective effect observed after vaccination in the lower respiratory tract. Although increased rates of fecal shedding for JN.1 were observed in recent wastewater surveillance data^36^, neither marked fecal excretion nor enteric viral replication in mock-vaccinated hamsters, nor an effect of vaccination was observed. However, this might be explained by technical restrictions of cumulative fecal sampling techniques that do not reflect individual shedding.

Assessing the humoral immune responses in the hamster after vaccination, we observed a general increase in titers to the recently circulating variants after JN.1 vaccination, indicating that the vaccine accelerates antibody responses. However, KP.2 and KP.3.1.1 challenged vaccinated animals showed slight decrease in homologous neutralizing antibodies compared to the non-vaccinated group, which might be explained by immune imprinting. Degryse et al. observed high humoral immune responses to a primary antigen compared to challenge viruses in hamsters^37^, which corresponds to our observation that JN.1 titers were highest in all vaccinated animals that were challenged, regardless of challenge virus. Indicating that, similarly to humans, antigenic imprinting plays a role in adaptive immunity in the hamster.

Overall, our results indicate that relatively small antigenic shifts seen in the antigenic map are sufficient for a variant to replicate in the upper respiratory tract, granting the possibility of continuous circulation and spread in the population. In contrast, a bigger antigenic distance may be required to induce infection and disease induction in the lower respiratory tract. The combined analysis of antigenic properties of SARS-CoV-2 variants by antigenic cartography and experimental challenge studies provides essential data for future decision making in vaccine policies to update SARS-CoV-2 vaccines. Prospective studies investigating correlates of protection associated with vaccination that aim to predict the difference between disease, protection and transmission of SARS-CoV-2 ideally should also include cellular and mucosal immunity. In addition, it will be important to further investigate the added value of mucosal vaccinations to better protect the upper respiratory tract against newly emerging SARS-CoV-2 variants.

## Methods

### Ethics

Research involving animals was conducted in compliance with the Dutch legislation for the protection of animals used for scientific purposes (2014, implementing EU Directive 2010/63) and other relevant regulations. The licensed establishment where this research was conducted (Erasmus MC) has an approved Office of Laboratory Animal Welfare (OLAW) Assurance # F16-00046 (A5051-01). Research was conducted under a project license (#2317528) from the Dutch competent authority and the study protocol was approved by the institutional Animal Welfare Body (Erasmus MC). During the vaccination period, animals were housed in individually ventilated cages (IVC green line, Techniplast) in groups of 3 animals. From one week prior to challenge with SARS-CoV-2, animals were housed in groups of 3 animals in filter top cages (Type 3, Techniplast), in Class III isolators, allowing social interactions, under controlled conditions of humidity, temperature and light (12-h light/12-h dark cycles). Food and water were available ad libitum. Animals were cared for and monitored (pre and post inoculation) daily by qualified personnel. All animals were allowed to acclimatize to husbandry for at least 7 days. For unbiased experiments, all animals were randomly assigned to experimental groups. The animals were anesthetized (3% isoflurane) for all invasive procedures. Hamsters were euthanized by cardiac puncture under isoflurane anesthesia.

### Cell lines

Calu-3 cells (ATCC HTB-55; RRID:CVCL_0609) were cultured in Opti-MEM (1x) + GLutaMAX (Gibco) supplemented with 10% FBS, penicillin (100 IU/mL), and streptomycin (100 IU/mL) at 37 degrees Celsius in a humidified CO_2_ incubator. When plating cells in 96-wells plates, the medium was supplemented with 10 mM Y-27632 apoptosis inhibitor (MedChemExpress). Cells were tested regularly for mycoplasma.

### Viruses

Viruses used are identified in Table 1. SARS-CoV-2 EG.5.1.1 and BA.2.86.1 were propagated on VeroE6-TMPRSS2 cells up to passage 3 and 2, respectively, and the consecutive passage was grown on Calu-3 cells. All other variants were isolated on either Calu-3 cells or human airway organoids at Air-Liquid-Interface and propagated to passage 3 on Calu-3 cells and were sequenced using nanopore of Illumina sequencing. P3 variants of SARS-CoV-2 Omicron JN.1, KP.2, and LP.8.1 were characterized as JN.1.55, KP.2.3, and LP.8.1.5 subvariants respectively.

**Table. 1:** Overview of SARS-CoV-2 virus strains used in this study

Virus strain	Identifier
SARS-CoV-2 (D614G)	OM304632
SARS-CoV-2 (Alpha)	MW947280
SARS-CoV-2 (Beta)	OM286905
SARS-CoV-2 (Gamma)	OM442897
SARS-CoV-2 (Zeta)	ON745569
SARS-CoV-2 (Delta)	OM287123
SARS-CoV-2 (Delta AY.4.2)	ON545851
SARS-CoV-2 (Lambda)	ON545854
SARS-CoV-2 (Mu)	ON479433
SARS-CoV-2 (Omicron BA.1)	OM287553
SARS-CoV-2 (Omicron BA.2)	ON545852
SARS-CoV-2 (Omicron BA.5)	isolate hCov-19/Netherlands/ZH-EMC-6149
SARS-CoV-2 (Omicron BM.1.1.1)	isolate hCov-19/Netherlands/ZH-EMC-6674
SARS-CoV-2 (Omicron BQ.1.1)	isolate hCov-19/Netherlands/ZH-EMC-7095
SARS-CoV-2 (Omicron XBB.1)	isolate hCov-19/Netherlands/ZH-EMC-7091
SARS-CoV-2 (Omicron XBB.1.5)	isolate hCoV-19/Netherlands/NH-EMC-5667
SARS-CoV-2 (Omicron XBB.1.9)	isolate hCov-19/Netherlands/ZH-EMC-7948
SARS-CoV-2 (Omicron XBB.1.16)	isolate hCov-19/Netherlands/ZH-EMC-8086
SARS-CoV-2 (Omicron EG.5.1.1), kindly provided by Dr. Etienne Simon-Loriere	isolate hCoV-19/France/GES-IPP15954/2023,
SARS-CoV-2 (Omicron BA.2.86.1), kindly provided by Dr. Etienne Simon-Loriere	isolate hCoV-19/France/IDF-IPP17625/2023
SARS-CoV-2 (Omicon JN.1)	isolate hCov-19/Netherlands/NB-EMC-5381
SARS-CoV-2 (Omicron KP.2)	isolate hCov-19/Netherlands/ZH-EMC-8727
SARS-CoV-2 (Omicron KP.3.1.1)	isolate hCov-19/Netherlands/ZH-EMC-8841
SARS-CoV-2 (Omicron XEC)	isolate hCov-19/Netherlands/ZH-EMC-8908
SARS-CoV-2 (Omicron LP.8.1)	isolate hCov-19/Netherlands/ZH-EMC-9114

### Focus reduction neutralization assay

For the neutralization assays, an 8-h infectious titer was determined. Stocks were thawed and 10-fold serially diluted in Opti-MEM (1x) + GLutaMAX (Gibco) and penicillin (100 IU/ml), and streptomycin (100 IU/ml). 100 µl of each dilution was added to a 96 wells plate containing confluent Calu-3 cells and incubated for 8 hs at 37 degrees. After 8 hs plates were fixed with formalin and permeabilized with ethanol. Plates were stained and positive cells were quantified as described in the following paragraph.

To determine the IC_50_ FRNT titers, sera were heat inactivated at 56 degrees Celsius for 30 min. A 2- or 3- fold serial dilution of serum samples was made in 60 uL Opti-MEM (1x) + GlutaMAX (Gibco) supplemented with penicillin (100 IU/mL), and streptomycin (100 IU/mL). Four hundred plaque-forming units of virus, as determined by an 8-h infectious virus titration, were diluted in Opti-MEM (1x) + GlutaMAX (Gibco) supplemented with penicillin (100 IU/mL), and streptomycin (100 IU/mL) to the volume of 60 µl. The virus dilution was added to the serial dilution of serum and incubated 1 h at 37 °C. Next, 100 µl of the virus-serum mix was added to confluent monolayers of Calu-3 cells grown in 96 well flat-bottom cell culture plates (Greiner) and incubated for 8 h at 37°. After 8 h plates were fixed in formalin and permeabilized with ethanol. After permeabilization with ethanol, plates were washed in PBS. Plates were stained with SARS-CoV-2 Nucleocapsid Rabbit (Sino Biological; RRID:AB_2892769; 1:5000) in PBS containing 0.5% BSA (bovine serum albumin; Sigma-Aldrich) and incubated 1 h at room temperature. After 1 h, plates were washed in PBS and stained with goat anti-rabbit Alexa Fluor 488 (Invitrogen; RRID:AB_2633280; 1:5000) in PBS containing 0.5% BSA and left to incubate for 1 h at room temperature. Nuclei were stained with Hoechst (Thermo Fisher Scientific; 1:1000) for 15 min. Plates were imaged with the Opera Phenix spinning disk confocal HCS system (PerkinElmer) or Amersham Typhoon and analyzed using Harmony software or ImageQuant TL software, respectively, to quantify positive cells. IC_50_ values were calculated using GraphPad PRISM 10 software.

### Antigenic cartography

The antigenic map was created from FRNT_50_ data, as described previously^10^. Briefly, to construct the antigenic map, the fold changes in neutralization titers from the maximum titer variant are calculated for each antiserum-antigen pair, representing the table distance. Subsequently, antiserum-antigen Euclidean map distances are optimized to ensure that the map distance accurately reflects the table distance, and the map stress (error between table and map distance) is minimized. One unit on the antigenic map represents a 2-fold change in neutralization titers. The map was made using the Racmacs package (https://acorg.github.io/Racmacs, version 1.2.9) in R (version 4.4.2). Utilizing the ‘make.acmap’ function, with 10000 optimizations and setting the minimum column basis parameter to ‘none’. Additional analyses were conducted with the ‘dimensionTestMap’ and ‘triangulationBlobs’ functions.

### Animals and experimental setup

Male Syrian golden hamsters (Mesocricetus auratus; 6 weeks old; Janvier, France) were handled in an ABSL-2 (vaccination period) and ABSL-3 biocontainment laboratory (challenge period), respectively. One group of animals (n = 36) was immunized intramuscularly in the hind leg (M. quadriceps) with 5 µg of stabilized SARS-CoV-2 JN.1 S trimer (Acro Biosystems) reconstituted in sterile deionized water with 2% Alhydrogel (InvivoGen) as an adjuvant at a 1:1 ratio in a final volume of 100 µl. A second group of animals (n = 36) served as mock-vaccinated control and was immunized with sterile deionized water with 2% Alhydrogel at a 1:1 ratio in a final volume of 100 µl. After 21 days, animals were boosted in the same composition as the first vaccination. The approved protocol did not allow blood sampling before challenge. Twenty-one days after the booster vaccination, groups of JN.1-and mock-vaccinated animals (n = 6) were challenged with an intranasal inoculation of SARS-CoV-2 JN.1 (1.0 × 10^4^ PFU/animal), KP.2 (1.0 × 10^4^ PFU/animal) or KP.3.1.1 (6.0 × 10^3^ PFU/animal) in a total volume of 100 µl per animal. Throat swabs and collective feces samples per cage were taken at 1, 3, 5, 7, 10 and 14 days post inoculation (dpi). The body weight of animals was measured daily. On 4dpi, animals (n = 6) from each experimental group were euthanized and the respiratory tract (nasal turbinate, trachea and lungs) as well as extra-respiratory tissue (olfactory bulb, small intestine, large intestine) was sampled for quantification of viral and genomic load, as well as for histopathology and virus antigen expression. All remaining animals were sacrificed 14dpi.

### Viral titrations

Plaque assay titrations of throat swabs and tissue samples from 4 dpi were performed by thawing samples and making a 10-fold serial dilution in Opti-MEM (1x), penicillin (100 IU/ml), and streptomycin (100 IU/ml). 100 µL of each dilution was added to 24 wells plates containing fully confluent monolayers of Calu-3 cells in the same medium. After a 4 h incubation at 37°C, the inoculum was replaced with 1.2% Avicel (FMC biopolymers) in Opti-MEM (1x), penicillin (100 IU/ml), and streptomycin (100 IU/ml). Cells were incubated for 1-2 days, fixed with formalin and permeabilized with 70% ethanol. After permeabilization, plates were washed in PBS and blocked with PBS containing 0.5% BSA for 30 min. The primary antibody SARS-CoV-2 Nucleocapsid Rabbit (Sino Biological; RRID:AB_2892769; 1:5000) was added in PBS containing 0.5% BSA and incubated 1 h at room temperature. Plates were washed in PBS and the secondary goat anti rabbit Alexa Fluor 488 antibody (Invitrogen; RRID:AB_2633280; 1:5000) was added in PBS containing 0.5% BSA for 1 h at room temperature. After a final wash with PBS, plates were imaged on the Amersham Typhoon followed by analysis using ImageQuant TL software.

### RNA extraction and genome quantification with RT-qPCR

All throat swabs, feces homogenates and tissue samples were used for RNA extraction and following quantification of SARS-CoV-2 genome by RT-qPCR. RNA extraction was performed as described previously^38^. A RT-qPCR targeting the E gene of SARS-CoV-2 was used as previously reported^39^. Ct values were compared to a standard curve derived from a titrated JN.1 virus stock. Additionally, lungs of all animals sacrificed on 4dpi were stored in RNAlater (Thermo Fisher) and homogenates were transferred to TRIzol (Invitrogen) and RNA was extracted according to manufacturer’s instructions. Host genes (IL6, IL1ß, TNFα, IFNγ and CXCL10) were measured utilizing hamster specific primers and probes^40^ and were normalized to the housekeeping gene ß-actin in a duplex RT-qPCR.

### Histopathology and immunohistochemistry

Heads, trachea, lungs, small- and large intestines were fixed in 4% neutral-buffered formalin, embedded in paraffin, and sectioned at 3 µm. Sections of all tissue samples were stained with haematoxylin and eosin for histopathological analysis, and consecutive sections were stained by immunohistochemistry for SARS-CoV-2 antigen expression, as described previously^41^. Immunohistochemistry of heads and lungs were scanned with the Hamamatsu Nanozoomer 2.0 HT digital slide scanner and relative positive cells in lungs were quantified via a positive cell detection threshold in QuPath Open software version 0.5.1. For visualization in shown figures, contrast and brightness of representative images was adjusted. Dust particles and reflections, as well as remaining parts of cardiac tissue were removed from whole slide scan visualizations for Fig. 4.

### Statistical analysis

All statistical analyses were performed using GraphPad Prism 10 software (La Jolla). Applied tests are indicated in according figure legends.

### Role of the funding source

Study sponsors had no role in study design; in the collection, analysis, and interpretation of data; in the writing of the report; and in the decision to submit the paper for publication.

### Resource availability

#### Lead contact

Requests for further information and resources should be directed to and will be fulfilled by the lead contact, Melanie Rissmann (m.rissmann@erasmusmc.nl).

## Materials availability

This study did not generate new unique reagents.

## Supplementary information

2025-11-11_Supplementary Figures_complete

## Data Availability

All original data are presented in main and supplemental figures or are available upon request from the corresponding author. This study did not generate standardized data types. This paper does not report original code. Any additional information required to reanalyze the data reported in this paper is available from the corresponding author upon request.
