# Supplementary material for: Assessment of SARS-CoV-2 immune escape using antigenic cartography combined with experimental challenge studies
Source: NPJ Vaccines. 2026 Jan 2;11:25. doi: 10.1038/s41541-025-01348-x (PMC12847947; doi:10.1038/s41541-025-01348-x)
Supplement: Supplementary file 1 — 2025-11-11_Supplementary Figures_complete [file 41541_2025_1348_MOESM1_ESM.pdf]

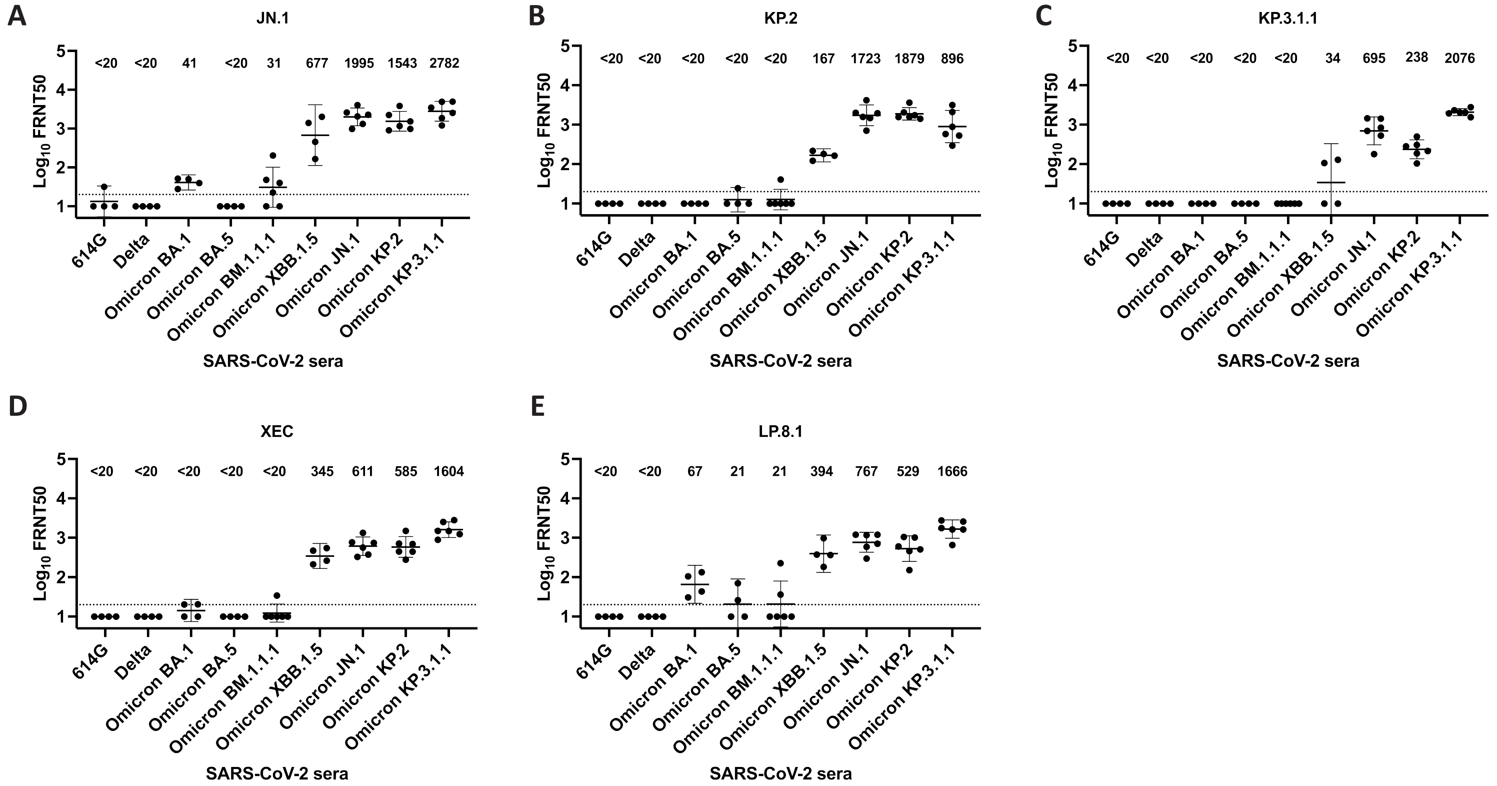

Supplementary Figure 1. Neutralizing titers against recently circulating SARS-CoV-2 variants

(A) JN.1, **(B)** KP.2, **(C)** KP.3.1.1, **(D)** XEC and **(E)** LP.8.1 $FRNT_{50}$ neutralizing antibody titers were tested in hamster sera generated against viruses indicated on the X-axis. Geometric mean titers with 95% CI are shown and indicated above. The dotted line represents the lower limit of detection.

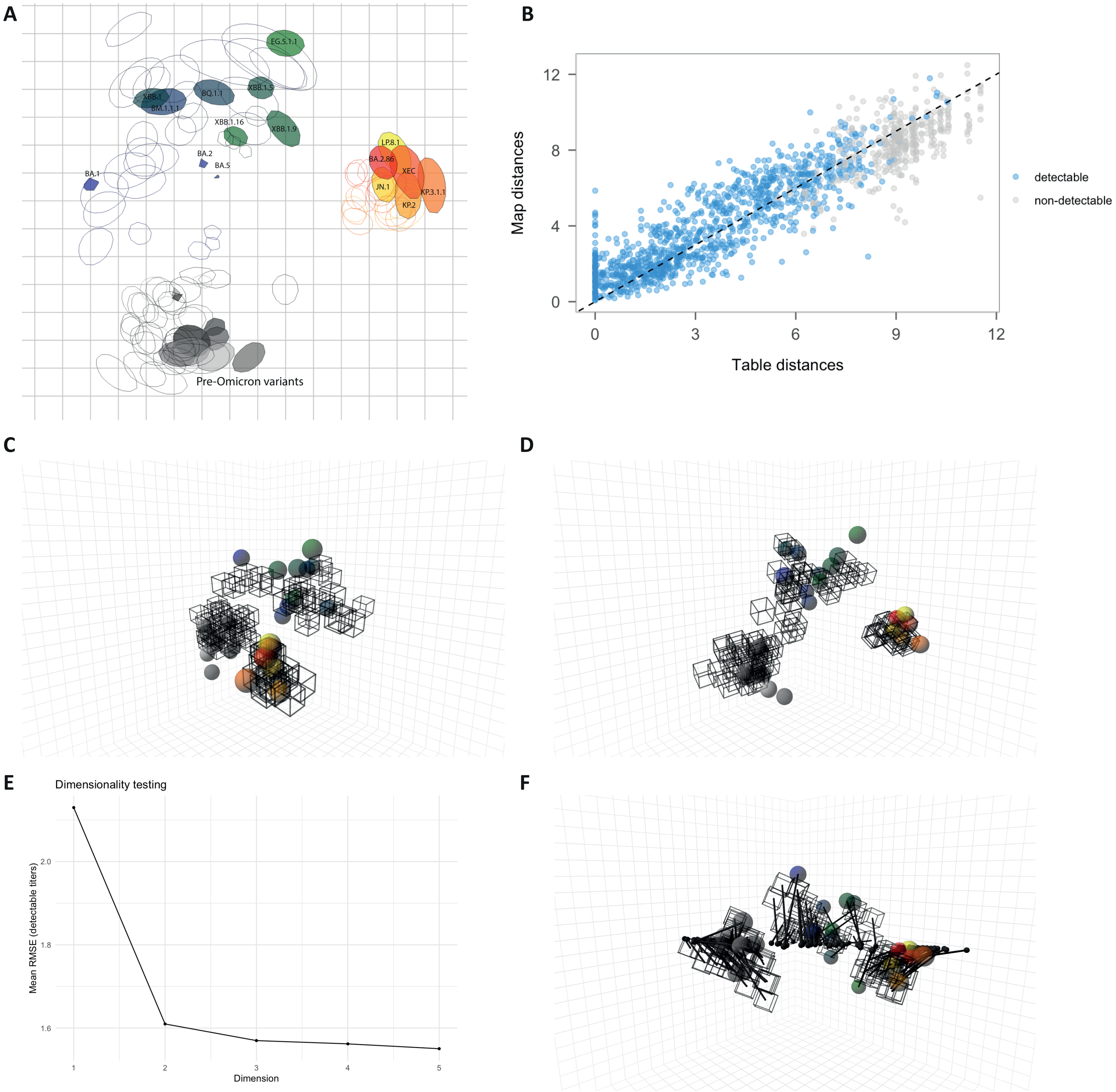

Supplementary Figure 2. Antigenic map validation and 3D projection

(A) Antigenic map from Figure 1, whereby each region (triangulation blob) represents the space where each serum and variant can position without increasing map stress by more than one unit. **(B)** Correlation between Table distance (obtained from FRNT) and Map distance. **(C-D)** Three-dimensional view of the antigenic map shown from two different perspectives. **(E)** Dimensionality test indicating the mean root mean square error (RMSE) of detectable neutralizing titers. **(F)** A 3D antigenic map connecting the positions of antigen and antisera to a two-dimensional plane.

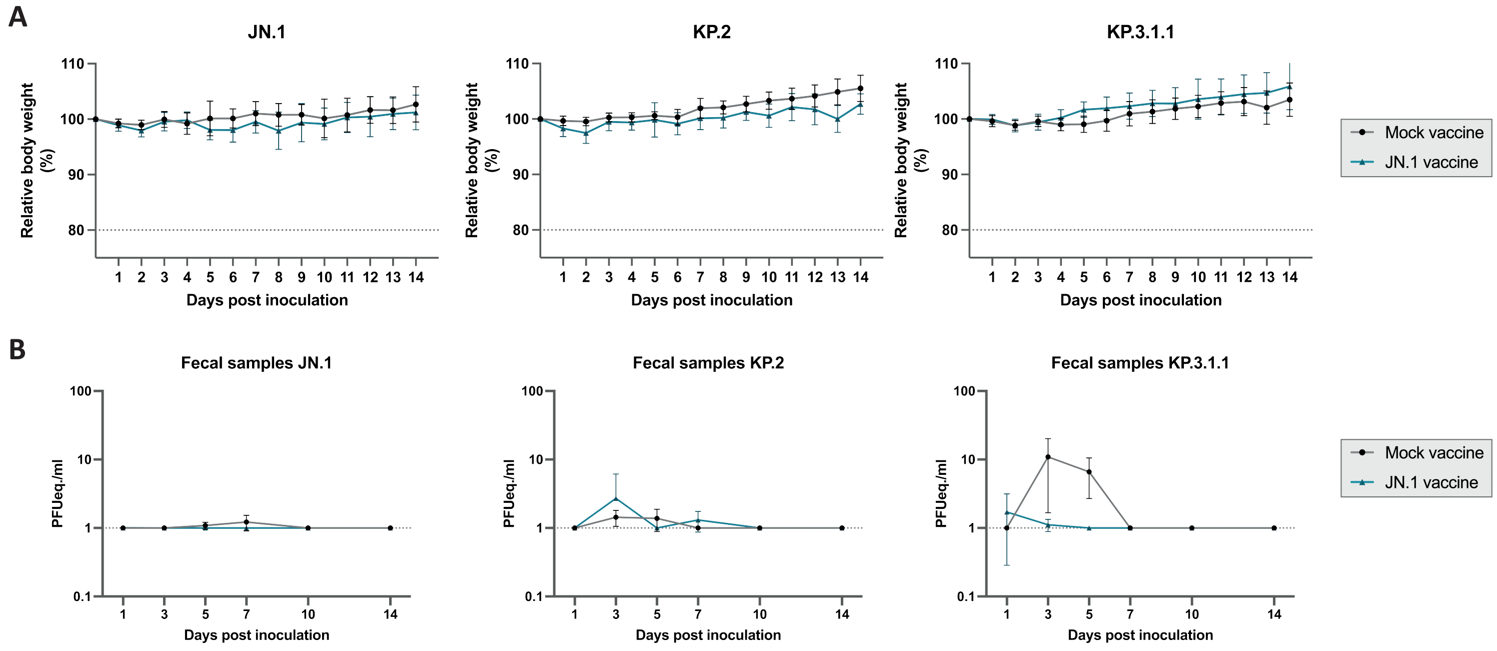

Supplementary Figure 3. JN.1 vaccine-induced effects *in vivo*

(A) Progression of relative body weight over a course of 14 dpi. Dotted line indicates the humane endpoint of maximum body weight loss. **(B)** qRT-PCR results of bulk fecal samples that were collected for each cage. Results are depicted in PFUeq./ml. Statistical significant differences were measured by Mann-Whitney test with multiple comparisons according to the Holm-Šídák method. Error bars represent interquartile range.

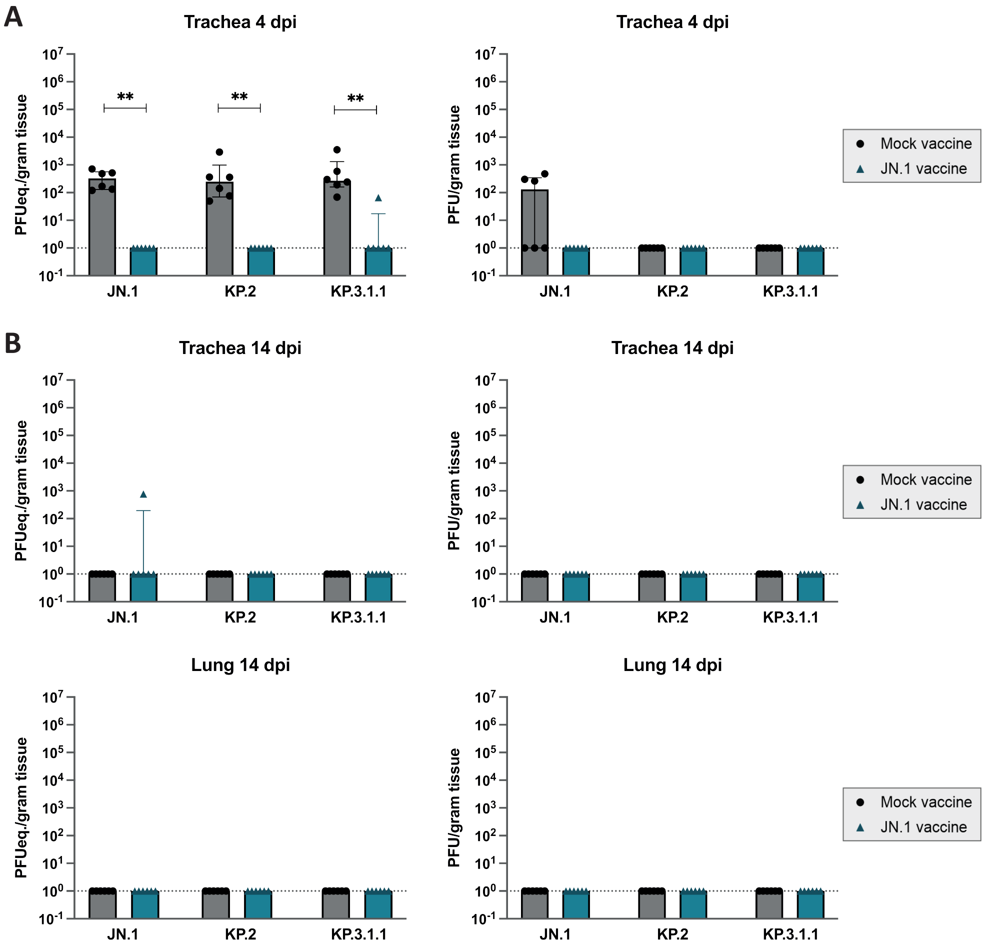

Supplementary Figure 4. Viral titration of lower respiratory tract tissues

qRT-PCR results of respiratory tissue homogenates from **(A)** trachea 4 dpi and **(B)** trachea and lung 14 dpi, depicted in PFUeq./g tissue and results of plaque assay to determine infectious virus in PFU/g tissue. Statistical significant differences were measured by Mann-Whitney test. Error bars represent interquartile range. Dotted lines represent the lower limit of detection and symbols represent individual measurements for each animal. Six animals were included per experimental condition. *p<0.05, **p<0.005.

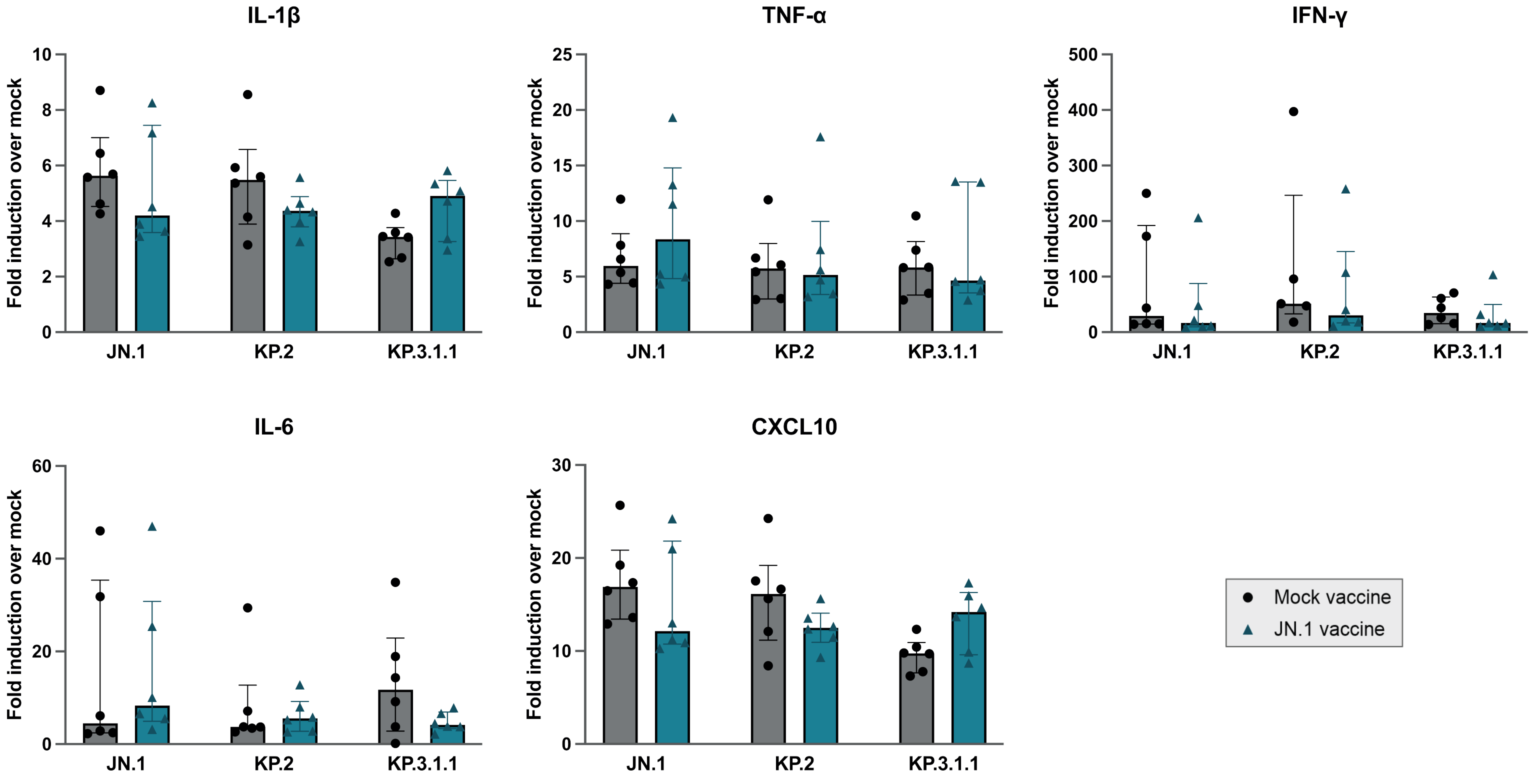

Supplementary Figure 5. JN.1 vaccine-induced effects on host gene expression in lungs

qRT-PCR results of host gene expression. Values of challenged animals were compared to a historical, non-infected control group and with the ΔΔCt-method, the fold-change induction was calculated. Symbols represent individual measurements for each animal. Six animals were included per experimental condition.

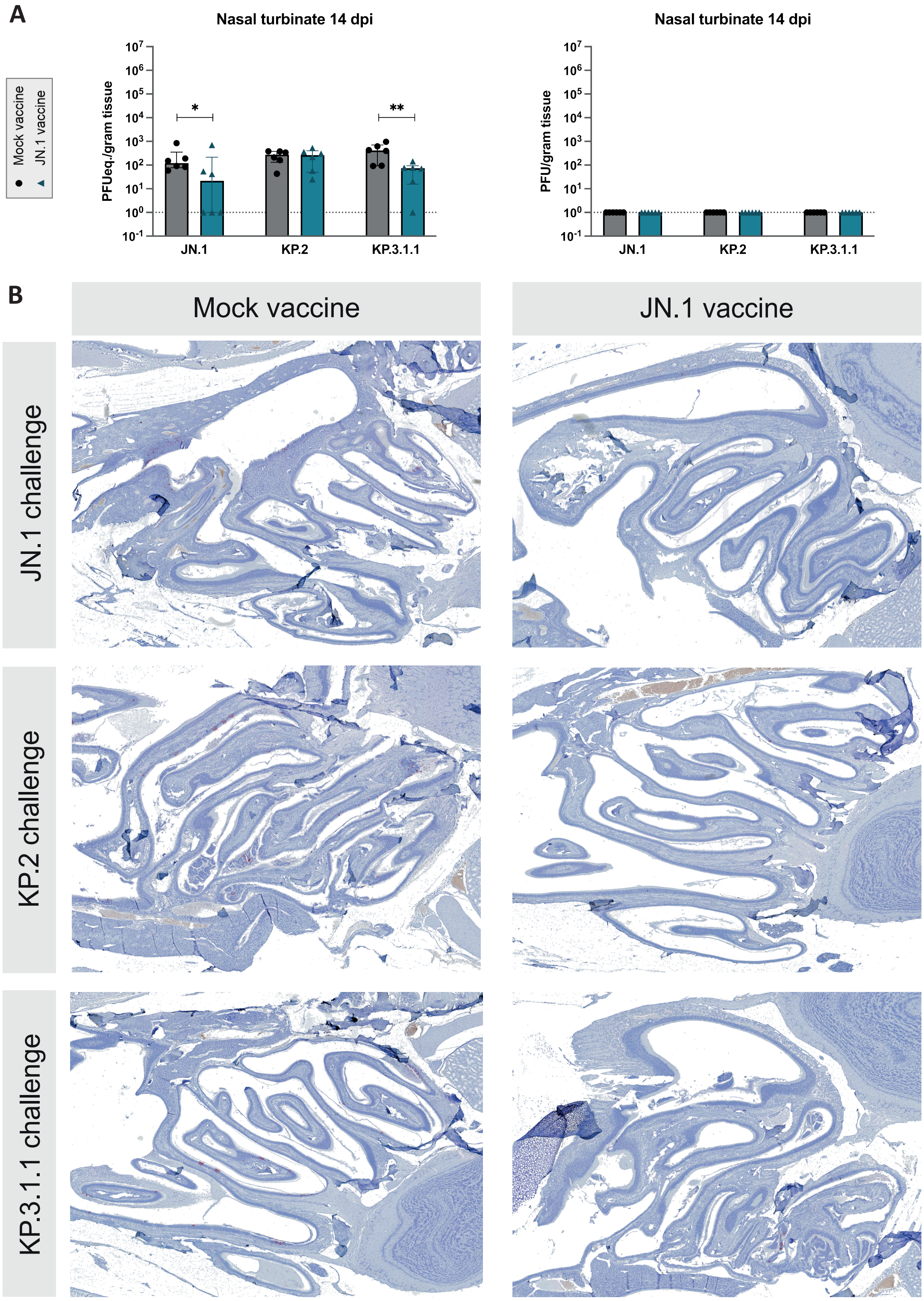

Supplementary Figure 6. JN.1 vaccine-induced effects in the upper respiratory tract

(A) qRT-PCR results of nasal turbinate 14 dpi, depicted in PFUeq./g tissue and results of plaque assay to determine infectious virus in PFU/g tissue. Statistical significant differences were measured by Mann-Whitney test. Error bars represent interquartile range. Dotted lines represent the lower limit of detection and symbols represent individual measurements for each animal. Six animals were included per experimental condition. $*p<0.05$, $**p<0.005$. **(B)** Histopathology panel showing immunohistochemistry (IHC) of the nasal turbinate at 4 dpi of mock-vaccinated hamsters compared to JN.1-vaccinated hamsters after intranasal challenge with one of three SARS-CoV-2 variants JN.1, KP.2, and KP.3.1.1, from top to bottom respectively. Virus antigen is expressed as reddish-brown staining by AEC immunoperoxidase, and hematoxylin counterstain.

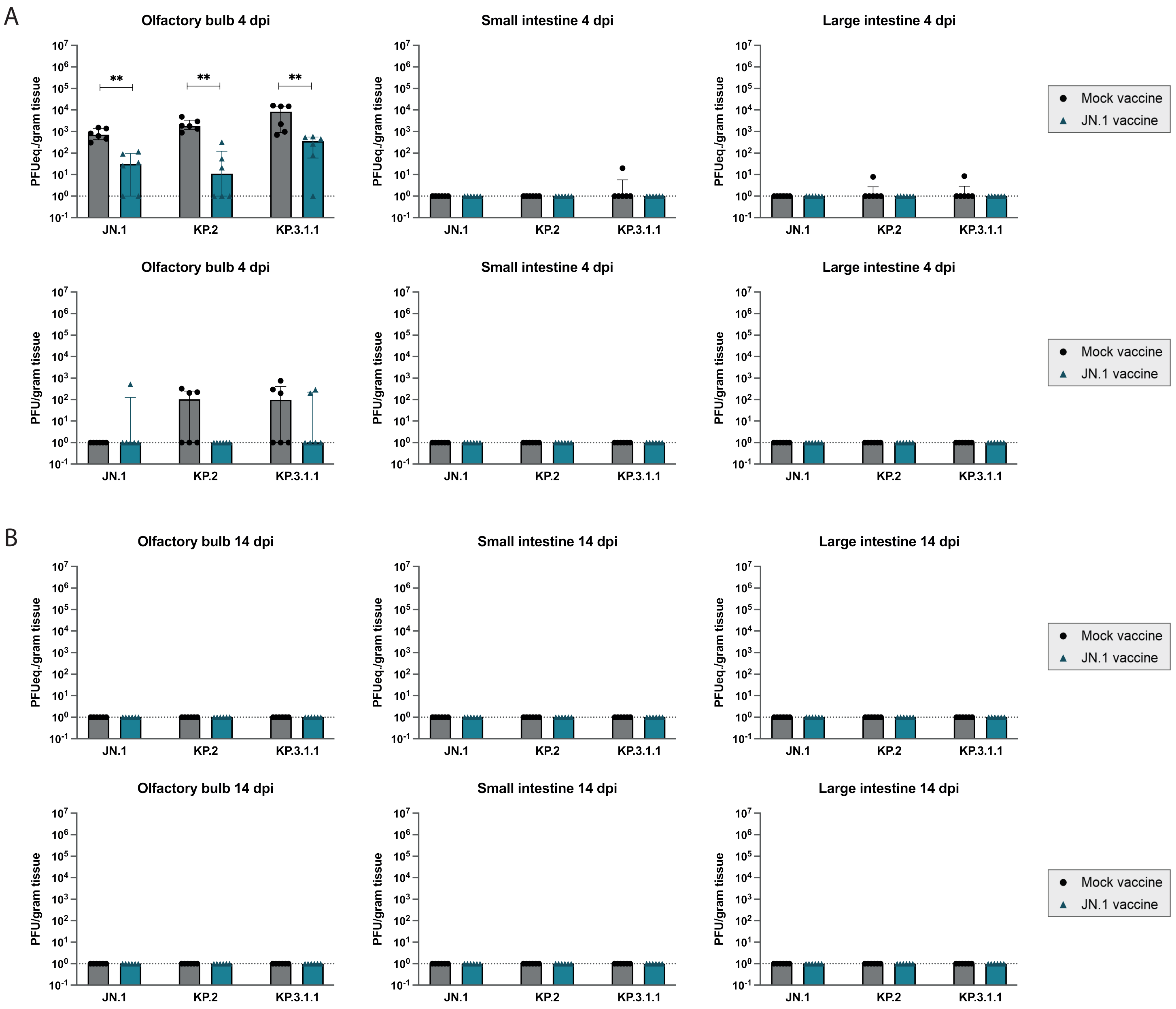

Supplementary Figure 7. JN.1 vaccine-induced effects on extra respiratory tissues

(A) qRT-PCR results of extra respiratory tissue homogenates from 4 dpi, depicted in PFUeq./g tissue and results of plaque assay to determine infectious virus in PFU/g tissue. Statistical significant differences were measured by Mann-Whitney test. Error bars represent interquartile range. **(B)** qRT-PCR results of extra respiratory tissue homogenates from 14 dpi, depicted in PFUeq./g tissue and results of plaque assay to determine infectious virus in PFU/g tissue. Statistical significant differences were measured by Mann-Whitney test. Error bars represent interquartile range. Dotted lines represent the lower limit of detection and symbols represent individual measurements for each animal. *$p<0.05$, **$p<0.005$. Six animals were included per experimental condition.

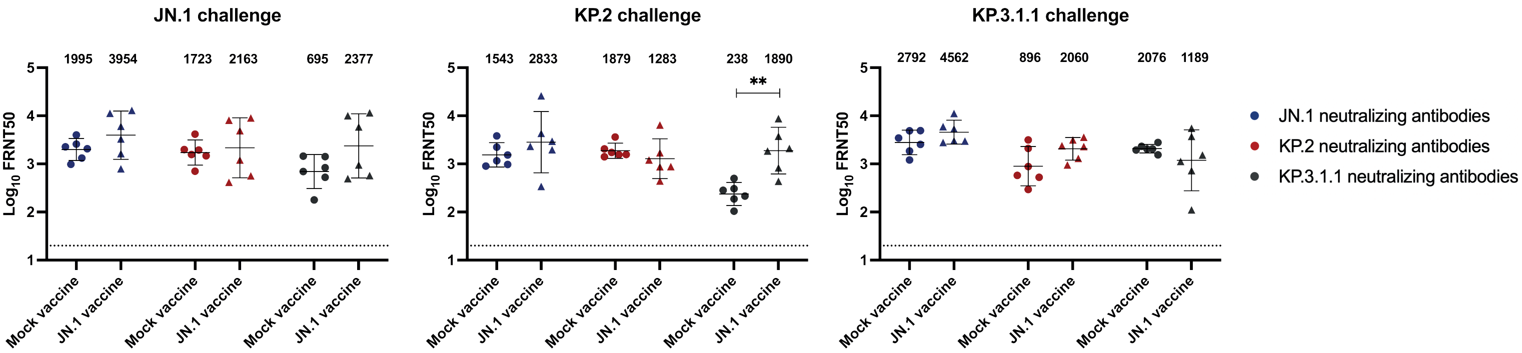

Supplementary Figure 8. Effect of JN.1 vaccination on neutralizing antibody titers

$FRNT_{50}$ neutralizing antibody titers were determined in hamster sera 14 dpi against JN.1, KP.2, and KP.3.1.1. The geometric mean is displayed above the graph, error bars indicate 95% confidence interval. Statistical significant differences were measured by Mann-Whitney test. Dotted lines represent the lower limit of detection and symbols represent individual measurements for each animal. *p<0.05, **p<0.005. Six animals were included per experimental condition.

	Mock vaccinated, JN.1 challenge	JN.1 vaccinated, JN.1 challenge	Mock vaccinated, KP.2 challenge	JN.1 vaccinated, KP.2 challenge	Mock vaccinated, KP3.1.1 challenge	JN.1 vaccinated, KP.3.1.1 challenge
**Severity alveolitis** (0-3)	**1** (1, 1, 1, 0, 2, 1)	**0.3** (0, 0, 0, 1, 0, 1)	**0.8** (1, 0, 1, 1, 1, 1)	**0.8** (0, 1, 1, 1, 1, 1)	**1.7** (1, 2, 1, 2, 2, 2)	**1** (1, 0, 2, 1, 1, 1)
**Presence edema alveolar / perivascular** (0/1)	**1** (1, 1, 1, 1, 1, 1)	**0.3** (0, 0, 0, 1, 1, 0)	**1** (1, 1, 1, 1, 1, 1)	**0.5** (0, 1, 1, 0, 1, 0)	**1** (1, 1, 1, 1, 1, 1)	**0.7** (1, 0, 1, 0, 1, 1)
**Presence alveolar haemorrhage** (0/1)	**0.5** (0, 0, 0, 1, 1, 1)	**0.2** (0, 0, 0, 0, 1, 0)	**0.2** (0, 0, 1, 0, 0, 0)	**0.5** (0, 1, 1, 0, 1, 0)	**0.7** (0, 1, 0, 1, 1, 1)	**0.5** (0, 0, 1, 0, 1, 1)
**Presence type II hyperplasia** (0/1)	**0** (0, 0, 0, 0, 0, 0)	**0** (0, 0, 0, 0, 0, 0)	**0** (0, 0, 0, 0, 0, 0)	**0** (0, 0, 0, 0, 0, 0)	**0** (0, 0, 0, 0, 0, 0)	**0** (0, 0, 0, 0, 0, 0)
**Severity bronch(iol)itis** (0-3)	**0.8** (1, 1, 1, 0, 1, 1)	**0** (0, 0, 0, 0, 0, 0)	**0.8** (1, 0, 1, 1, 1, 1)	**0.2** (0, 0, 0, 0, 1, 0)	**1.3** (1, 2, 1, 1, 2, 1)	**0.3** (0, 0, 1, 0, 0, 1)
**Degree perivascular cuffing** (0-3)	**1.3** (1, 2, 1, 0, 2, 2)	**0.3** (0, 0, 1, 0, 0, 1)	**1.2** (1, 0, 2, 2, 1, 1)	**0.3** (0, 0, 1, 0, 0, 1)	**1.7** (1, 2, 1, 2, 2, 2)	**0.2** (0, 0, 1, 0, 0, 0)
**Severity lung vasculitis (diapedesis)** (0-3)	**1.2** (2, 1, 1, 0, 2, 1)	**0.3** (0, 0, 1, 0, 0, 1)	**1.7** (2, 0, 2, 2, 2, 2)	**0.3** (0, 0, 1, 0, 0, 1)	**1.8** (2, 2, 1, 2, 2, 2)	**0.2** (0, 0, 1, 0, 0, 0)
**Severity tracheitis** (0-3)	**0** (0, 0, 0, 0, 0, 0)	**0** (0, 0, 0, 0, 0, 0)	**0** (0, 0, 0, 0, 0, 0)	**0** (0, 0, 0, 0, 0, 0)	**0** (0, 0, 0, 0, 0, 0)	**0** (0, 0, 0, 0, 0, 0)
**IHC trachea** (0-3)	**0** (0, 0, 0, 0, 0, 0)	**0** (0, 0, 0, 0, 0, 0)	**0** (0, 0, 0, 0, 0, 0)	**0** (0, 0, 0, 0, 0, 0)	**0** (0, 0, 0, 0, 0, 0)	**0** (0, 0, 0, 0, 0, 0)
**Small intestine / IHC** (0-3)	**0** (0, 0, 0, 0, 0, 0)	**0** (0, 0, 0, 0, 0, 0)	**0** (0, 0, 0, 0, 0, 0)	**0** (0, 0, 0, 0, 0, 0)	**0** (0, 0, 0, 0, 0, 0)	**0** (0, 0, 0, 0, 0, 0)
**Large intestine / IHC** (0-3)	**0** (0, 0, 0, 0, 0, 0)	**0** (0, 0, 0, 0, 0, 0)	**0** (0, 0, 0, 0, 0, 0)	**0** (0, 0, 0, 0, 0, 0)	**0** (0, 0, 0, 0, 0, 0)	**0** (0, 0, 0, 0, 0, 0)

Supplementary Table 1. Histopathological scoring of respiratory and enteric tissue

Tissues from 4 dpi were stained in HE and for viral antigen (n=6 per experimental group). Slides were evaluated by a trained veterinary pathologist. Semiquantitative scoring was performed, with ranking from 0-3 (0=non, 1 = mild; 2 = moderate; 3 = severe) or 0-1 (0 = not evident; 1 = evident).
